# Biological activities of *Ficus carica* latex for potential therapeutics in Human Papillomavirus (HPV) related cervical cancers

**DOI:** 10.1038/s41598-018-37665-6

**Published:** 2019-01-31

**Authors:** Arshia Ghanbari, Adam Le Gresley, Declan Naughton, Nikolai Kuhnert, Diana Sirbu, G. Hossein Ashrafi

**Affiliations:** 1 Kingston University London, Cancer theme, School of Life Science, Pharmacy and Chemistry, SEC Faculty, Kingston upon Thames, KT1 2EE London, UK; 2 Kingston University London, School of Life Science, Pharmacy and Chemistry, SEC Faculty, Kingston upon Thames, KT1 2EE London, UK; 3 Department of Life Sciences & Chemistry Faculty of Health, Jacobs University Bremen, Campusring 8, 28759 Bremen, Germany

## Abstract

Infection caused by high-risk human papillomaviruses (HPVs) are implicated in the aetiology of cervical cancer. Although current methods of treatment for cervical cancer can ablate lesions, preventing metastatic disseminations and excessive tissue injuries still remains a major concern. Hence, development of a safer and more efficient treatment modality is of vital importance. Natural products from plants are one of the principal sources of precursors to lead compounds with direct pharmaceutical application across all disease classes. One of these plants is *Ficus carica*, whose fruit latex, when applied on HPV-induced skin warts, has shown potential as a possible cure for this virus related lesions. This study explores the *in vitro* biological activities of fig latex and elucidates its possible mechanisms of action on cervical cancer cell lines CaSki and HeLa positive for HPV type 16 and 18, respectively. Our data shows that fig latex inhibits properties that are associated with HPV-positive cervical cancer transformed cells such as rapid growth and invasion and substantially downregulated the expression of p16 and HPV onco-proteins E6, E7. These findings suggest *Ficus carica* latex has the potential to be used in the development of therapeutic modalities for the possible treatment, cure and prevention of HPV related cervical cancer.

## Introduction

Cancer is a major public health concern and is one of the leading causes of death worldwide^1^. Cervical cancer is one of the most common types of cancer, affecting women on a global scale^2^. Infection caused by high-risk human papillomaviruses (HPVs), especially type 16 and 18 are implicated in the aetiology of most cervical cancers^3^. Coupled with their involvement in cancer, these viruses can cause life-long debilitating diseases that may be accompanied by a significant negative impact on quality of life. High-risk HPV infections interfere with the molecular pathways that are responsible for regulating epithelial differentiation as well as cell proliferation^4,5^.

HPV onco-proteins E6 and E7 contribute towards cellular changes in HPV infected cells. These facilitate the persistence of infection that might allow the progression of the lesions towards cancer^6^. E6 interacts physically with tumour suppressor protein p53 and prevents its function; this activity will ultimately impede apoptosis. On the other hand, E7 binds to retinoblastoma (Rb) protein and prevents the interaction of Rb with its natural target, namely transcription factor E2F. Consequently the checkpoint that controls G1/S transition becomes distorted, causing uncontrolled proliferative lesions^7,8^.

Once proliferative lesions persist they can progress to high-grade ones and become an invasive form of cervical cancer^9,10^. It has been demonstrated that the presence of even minimal amounts of HPV DNA are associated with an increased risk in the development of cervical cancer^11^. Given the importance of cervical cancer, to date, there has been no satisfactory medical treatment for human papillomavirus related cervical cancer as most of the developed treatments (e.g., surgical excision, chemotherapy, and cryotherapy) are eventually accompanied by excessive tissue injury^12^. Therefore, there is a continuing demand for development of new strategies for treatment, which avoids tissue injury.

Herbal medicinal and biological studies have revealed that public interest in utilising traditional remedies has greatly increased^13–15^. Among the such medically relevant plants, the fig latex (*Ficus carica)*, when applied to low risk human papillomavirus (HPV) related skin warts and bovine papillomavirus (BPV) related warts, has shown potential as a possible cure for the virus without inevitable tissue injury and remedial complications^16–18^. Fig latex also reportedly offers various therapeutic effects such as anti-Herpes Simplex Virus (HSV)-1^19^, anti-bacterial activity^20^, anthelmintic^21^. As a consequence we investigated the biological activity of the *Ficus carica* latex on high risk HPV related cervical cancer. Herein, we show that *Ficus carica* latex effectively inhibits growth of HPV positive cervical cancer cells (CaSki and HeLa), without a cytotoxic effect on HPV and cancer-free human immortalised keratinocyte (HaCaT) cell line. The latex presents anti-cancer effects by various mechanisms, including induction of apoptosis and inhibition of cell transformation; colony formation, cell proliferation, migration and invasion. In addition to its potent anti-cancer effects, the results obtained indicate that Fig latex has profound influence on the deregulation of HPV oncoproteins (E6 and E7) and HPV diagnostic marker protein (p16) and initiates the reactivation of Rb and p53 tumor suppressor proteins. These findings provide insight into new therapeutic avenues against HPV-associated cervical cancers.

## Material and Methods

### Cell culture and cell lines

Cervical cancer cell lines positive for HPV type 16 (CaSki) and HPV type 18 (HeLa) and HPV free Human immortalised Keratinocytes (HaCaT) were used for this study. CaSki cells were maintained in Roswell Park Memorial Institute medium (RPMI-1640) (Sigma, UK), HeLa cells in Eagle’s Minimum Essential Medium (EMEM) (ATCC, UK) and HaCaT cells in Dulbecco’s Modified Eagle’s Medium (DMEM) (Life science, USA). All medias were supplemented with 10% Fatal Calf serum (FCS) (Sigma) and penicillin (100 U/ml; Sigma) and streptomycin (100 µg/ml; Sigma). Cell culture work was performed following strict aseptic techniques inside a laminar flow hood. All cells were incubated in a 5% CO_2_ incubator at 37 °C.

### Collection and purification of fig latex

**Figure 1:**
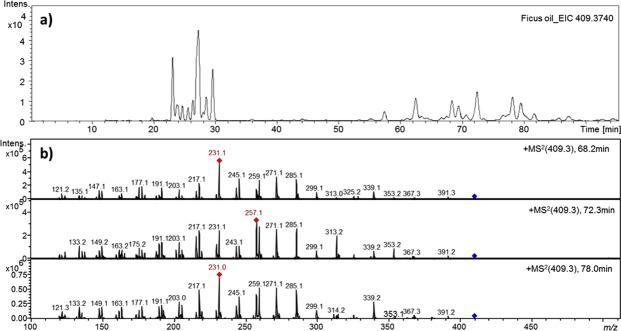
(**a**) Extracted ion chromatogram in positiv ion mode of *m/z* 409.3740 and (**b**) tandem mass spectrum showing the fragmentation of three isomers of *m/z* 409.3740 in Ficus oil extract.

Fig fruit latex was collected drop-by-drop without squeezing over summer months from unripe fruits of fig trees in the suburb of Tehran (Solughan-Iran) (Fig. 1) and 1 ml of the latex was put into in eppendorf tubes. Tubes were immediately stored at −20 °C until analysis. The latex was filtered using Whatman No. 1 (Fisher Scientific, UK) and centrifuged at 13000 rpm/4 °C to separate the polymeric gum from the aqueous filtrate part. Further purification of the aqueous part was subsequently attained by filtration using a 5 µm disposable filter membrane (Sigma, UK).

### Separation of fig latex supernatant extracts

Approx 40 mg of each crude Fig Latex sample was dissolved in 2 ml of a 90/10 v/v mixture of distilled water and D_2_O. The internal standard TSP (trimethylsilylpropionate sodium salt) was added to a concentration of 1.52 mM and each sample was shaken for a minimum of 8 min and solution confirmed, before transfer to an NMR tube. Each sample was allowed to equilibrate within the NMR spectrometer for 5 min. All NMR experiments were carried out at 25 °C. All crude spectra were acquired on Topspin 3.0 (BrukerBioSpin GmbH, Rheinstetten, Germany) and 64 K complex data points were acquired over a sweep width of 20.57ppm with a D1 relaxation delay of 4 s with water suppression afforded by the noesygppr1d sequence.

### Extraction of fig latex supernatant extracts

Fig latex supernatant (1 ml) was extracted into 3 × 5 ml Petroleum Ether 60/40. The residue was then extracted using 3 × 5 ml CHCl_3_. The final residue was taken up CH_3_CN. After the extractions, the individual organic extracts were combined and the solvents removed under reduced pressure. Each of the resulting powders was then assessed for cytotoxic activity.

### NMR Analysis

A Bruker Avance III 600 MHz NMR spectrometer with 5 mm TXI Probe and temperature control unit was used for all 1 H and 2D NMR experiments; 5-mm Bruker Single Use NMR tubes (Product Code Z117777) were used. All chemicals were sourced from Sigma-Aldrich and were used without further purification unless otherwise stated. The petroleum ether fraction of the fig latex supernatant was taken up in CDCl_3_ and ^1^H, ^13^C, ^1^H-^1^H COSY, ^1^H-^1^H TOCSY and ^1^H-^13^C HSQC experiments undertaken to aid in assigning the main types of components.

### MS Analysis

Ethanol gradient grade was purchased from Merck (Darmstadt, Germany), isopropanol (Rotisolv® HPLC grade), acetonitrile (Rotisolv® HPLC ultra gradient grade), chloroform (Rotisolv® HPLC grade) and Tetra-dodecylammonium bromide was purchased from Carl Roth (Karlsruhe, Germany), ammonium formate LC-MS Ultra and formic acid (puriss., ≥98% (T) for mass spectrometry), and acetic acid were purchased from Sigma-Aldrich Chemie (Steinheim, Germany). Ethanol was subjected to distillation prior use.

### Sample preparation

For HPLC analysis, a concentration of 0.045 mg/mL in chloroform/ethanol (50/50) of ficus oil extract was prepared.

### HPLC chromatographic conditions

Lipid molecular species were separated using an HPLC equipment (Agilent 1100 series, Waldbronn, Germany). The column used in this study was a Pursuit XRs C18 (250 mm × 3 mm i.d., 5 μm particles). The temperature of the column oven was set to 35 °C. 3 µL of sample were injected. Solvent A consisted of acetonitrile with 0.01% formic acid and solvent B consisted of ethanol with 10 mM/L ammonium formate and 0.01% formic acid. The mobile phase was pumped through the column at a flow rate of 0.6 mL/min. The gradient elution program consisted of holding solvent steady A (100) for 5 min; followed by a linear gradient to solvent A/B (70/30) for another 5; then by a linear gradient to solvent B (100) for 90 min, and ending with isocratic elution at solvent B (100) for 10 min. The column was equilibrated at 100 solvent A for 5 min before reuse.

### High-resolution mass spectrometry conditions

High-resolution mass values were acquired using a time of flight MicrOTOF Focus mass spectrometer (Bruker Daltonics UHT Ultra, Bremen, Germany) fitted with an ESI source used as the detector with the following parameter settings: capillary voltage of 4.5 kV; nebulizing gas pressure of 2 Ba; drying gas flow rate of 10 L/min; drying gas temperature of 220 °C. ESI mass spectra were measured in the range of m/z 200–1200 in the positive ion mode. Internal calibration was achieved with 10 mL of 0.1 M sodium formate solution injected through a six-port valve prior to each chromatographic run. Calibration was carried out using the enhanced quadratic mode.

### Tandem Mass spectrometry conditions

LC-tandem MS was carried out using an Ion-Trap detector in positive ion mode equipped with an ESI source (Bruker Daltonics UHT Ultra, Bremen, Germany). The full scan mass spectra were recorded in the range *m/z* 200–1200 operating in positive ion mode. Capillary temperature was set to 350 °C, drying gas flow rate of 10 L/min and nebulizer pressure of 10 psi. Tandem mass spectra were acquired in Auto MS^*n*^ (smart fragmentation) using a ramping of the collision energy.

### Cytotoxicity assay using MTT

In order to investigate the effect of fig latex on cell growth HPV expressing cervical cancer cells (CasKi and HeLa), and control HaCaT cells (4 × 10^3^) were seeded in 96 well plate. The following day, cells were treated with various concentrations (0.125, 0.25, 0.5, and 1 µg/ml) of fig latex. After 24, 48 and 72 hours, 20 $\mu l$ of MTT (Sigma, UK) solution 5 mg/ml in phosphate buffer solution (Thermofisher, UK) was added in to each well and plates were cultivated at 37 °C in 5% CO_2_ for 3–4 hours. Afterward, the supernatant was removed, the remaining formazan crystals were dissolved in 100 µl of DMSO and the absorbance was measured at 540 nm with an Epoch plate reader (BioTek, UK) considering wells without cells as blank. Obtained values were analysed with Gen5 software (BioTek, UK). The percent of proliferation in each treated cell line was normalised based on their control wells. All experiments were performed at least in triplicate.

### Colony forming assay

Cells were plated at approximately 600 cells/well in 2.5 ml media in 6-well corning plate and cultivated for 12 hrs to adhere. After that, cells were treated with concentrations of 0.125 µg/ml and 0.25 µg/ml of fig latex for period of 14 days. The cells were then washed 3 times in PBS (Thermo Fisher, UK), fixed in formaldehyde 3.7% (Sigma, UK), and stained with crystal violet (Sigma, UK). Visible colonies (~35–50 cells) were counted and presented as the ratio of the number of colonies in treated cells divided by the number of colonies in the control ones.

### Ki67 proliferation

For this assay, cells (1 × 10^3^) were seeded into 24-well plates containing coverslips. The following day, cells were treated with concentration of 0.125 $\mu$g/ml of fig latex for 48 hours. Afterward, cells were fixed in formaldehyde 3.7%, permeablisied in 0.1% triton-100 and blocked using BSA/PBS 3%. To detect Ki67 antibody, cells were incubated with Anti-ki67 Rabbit mAb (1:200) followed by Gout Anti-Rabbit (1:250). Cells were then washed in PBS 0.1% Tween-20 for five times and incubated with DAPI. The coverslips were then mounted onto slides. The images were captured using LEICA TCS SP scanner microscope and were analyzed by Leica Confocal Software (Heidelberg, Germany).

### Cell migration and invasion assays

Cell migration and invasion assays were performed using 24-well Transwell chamber with pore size 8 µm. 250 µl of cell suspension (50,000) without FCS was added into upper chamber and lower chamber was filled with medium containing 10% FCS as a chemoattractant. For cell invasion assay 250 µl of Extracellular Matrix (ThermoFisher) was added to lower chamber. 0.125 µg/ml of fig latex was added to upper chamber, cells were then cultivated at 37 °C for 24 hours in order to migrate and or invade from upper chamber towards lower one. Following that, cell from upper chamber removed gently with cotton swap, washed in PBS, fixed with formaldehyde 3.7% and stained with crystal violet 0.2%. All experiments were repeated at least three times. The number of cells was counted in 5 fields randomly and was scored based on their untreated wells.

### Cell Cycle Analysis

Cell cycle distribution was assesses by flow cytometry. Cells were treated with 0.125 $\mu$g/ml of fig latex or equivalent amount of PBS for 48 hrs. Approximately (1 × 10^6^) cells were harvested from both control and treated flasks. Cells were then washed in PBS and fixed in 70% ice-cold ethanol for 1 hr. Then 500 µl of PI/RNase (Thermofisher, UK) was added to samples and were kept in the dark for 20 minutes at 37 °C. Stained cells were then excited at 488 nm using the FL-3 detector (620 nM) of a BD FACsCalibur flow cytometer (Becton-Dickinson). Acquired data was analysed using CellQuest software (Becton-Dickinson).

### Western Blot

To investigate the effects of fig latex on HPV oncoproteins (E6 & E7), tumor suppressor proteins (P53 & Rb) and HPV infection surrogate marker (P16), standard semi-dry western blotting technique was used. Cells were treated with concentration 0.125 $\mu$g/ml of fig latex for 48 hrs then proteins were extracted using RIPA buffer (Life Technologies, UK) and equal amounts of protein were electrophoresed. Membranes were blocked with 5% (w/v) non-fat dry milk in TBS-T (10 mM Tris-HCl, pH 7.5, 150 mM NaCl, and 1% (v/v) Tween20) at room temperature for 2 hours to reduce non-specific binding and incubated with the mAb Anti-HPV16 E6 + HPV18 E6 antibody (1:500; Abcam), mAb Anti-HPV16 E7 (1:500; Abcam) + HPV18 E7 (1:750; Abcam) antibody, Anti P53 antibody (1:500; Abcam), Anti Rb Antibody (1:1000; Abcam) Anti p16 antibody (1:750; Abcam) followed by Donkey anti-mouse (1:10000; Abcam) and actin antibody (1:10000). The membrane was then visualised by OdysseyCLx Imaging System (*Li-COR)*.

### Statistical Analysis

Statistical analyses were performed using Microsoft Excel 2011 version (14.3.8). The correlations between cell viability and response to treatment were analysed using logarithmic regression of line. To calculate IC50 we obtained logarithmic regression of line (y = a ln (x) + b), using Excel. Since we were looking for inhibitory concentration for 50% of the cells, y value (axis) was equaled to 50. A and b values were given by excel therefore; calculation of x value was performed. X value represents concentration. Values were presented as mean standard error of the mean (S.E.M.). Statistical analyses were performed employing an unpaired, two-tailed student’s t-test. *p < 0.05 **p < 0.01, ***p < 0.001 were considered statistically significant, very significant and extremely significant.

## Results

### Chemical Analysis of *Ficus carica* Latex

Fig latex is a milky fluid comprising aqueous medium and gum that makes the latex sticky and solidifies on exposure to air. Direct application of milky fluid on cells as well as preparation of serial dilution for molecular biology studies was limited due to the stickiness of latex. To overcome this restriction and determine the active biological compartment of fig latex for therapeutic approaches, chemical components of lyophilized, aqueous part and polymeric gum of fig latex were compared using NMR and MS. Crude NMR analysis (see Supporting Information S1) revealed significant differences between the chemical constituents of the lyophilised latex and the aqueous part of the latex. Polymeric gum did not present significant chemical components. The components in the aqueous part of the latex are easier to discern than in as the crude latex (Lyophilised latex powder). The powder shows a mixture of saturated and unsaturated fatty acids/triglycerides. The aqueous part of latex was as effective as the whole latex on cells from a biological activity perspective. Therefore, the aqueous part of the latex (supernatant) was used and exclaimed as “latex” throughout of this study.

In order to identify the nature of the chemical species responsible for this activity, the fig latex supernatant was extracted, first with petroleum ether, with subsequent extractions performed with increasing polarity solvents. After drying, these extracts were tested for anti-HPV activity. As a consequence of the pet ether extract of the fig latex supernatant showing the most potent anticancer activity, a more detailed analysis was carried out. Initial LC-MS data (Fig. 1) indicated that there were many isomers present with a mass of 409, molecular formula being C_30_H_48_. The tandem MS was consistent with a cyclic terpene.

2D NMR analysis supports the MS, and also suggests that there are steroidal compounds present in the pet ether extraction of the fig latex supernatant. Specifically, there is also evidence for the related phytosterol fatty acid glycosides - 6-*O*-acyl-*β*-D-glucosyl-*β*-sitosterols. There is a precedent for this as reported by Rubnov^22^. What is also worthy of note is the presence of phenolic components, which in the Pet. ether fraction are easy to visualise via NMR in the aromatic region (See Supporting Information S2). Whilst the analysis is preliminary and further, more demanding, purification steps will be required to narrow down the many components to that one active, there is evidence of either two ferulic/caffeic/chlorogenic acid components in the pet ether fraction. In isolation these would be unlikely to partition well into the Pet. ether fraction from the fig latex supernatant, owing to their hydrophilicity, but if covalently attached to a sterol or cyclic terpene, these derivatives, whilst anticipated, have not yet been reported in fig latex as being responsible for any of the observed biological activity.

**Figure 2:**
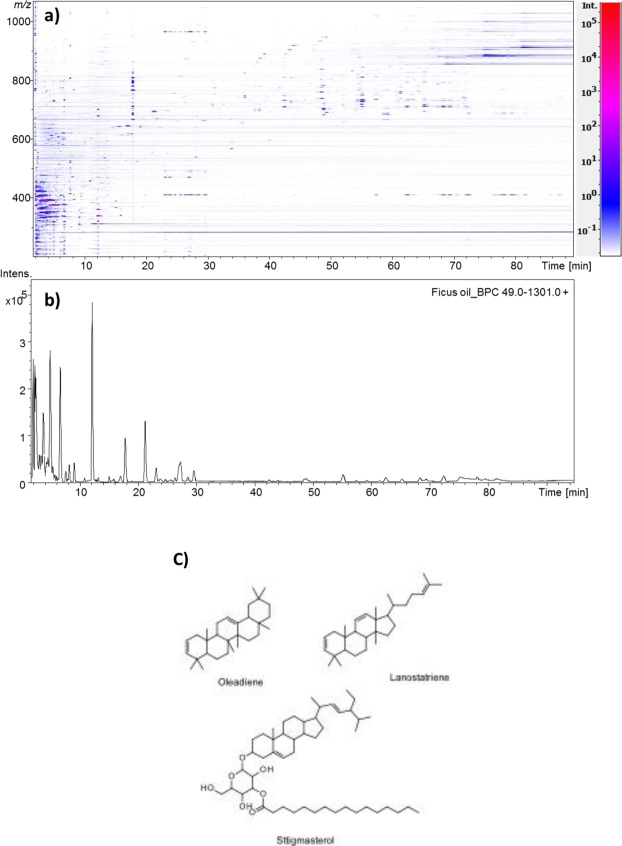
(**a**) Two-dimensional (2D) map of lipid of Ficus oil, by reversed-phase high resolution HPLC/ESI-MicroOTOF MS analysis. Colour rapresents the intensity of the signals (peaks) with red being the highest and blue the lowest and (**b**) HPLC-MS profile of Ficus oil in positive ionization mode. (**c**) MS determined structures of main chemical components of pet ether fraction of fig latex supernatant.

LC-ESI-MS chromatogram in positive ion mode showed that the most abundant lipid compounds present in the oil have the masses between 300 and 450 *m/z* ratio and elute at very low R_t_ (Fig. 2(a,b)). The low R_t_ indicates that the most abundant compounds have a polar nature. Besides, the same chromatograms showed the occurrence of few triacylglycerol molecular species and the presence of a compound with *m/z* value of 409.3840 as a mixture of various isomers eluting at different R_t_ covering a wide range between 19.5 min and 100 min (Fig. 2(a)). High-resolution ESI-TOF mass spectrometer operating in the positive ion mode lead to the assignment of molecular formulae of C_30_H_49_ with an error lower than 2 ppm. Tandem MS of the different isomers has shown similar fragmentation pattern resembling the tandem MS of a steroid compound. This supported the idea of oflanosteroltriene or oleandiene. This is supported by the NMR data. It was also observed that there were a few fatty acid glycosides of phytosterols e.g. stigmasterol, present in the supernatant (Fig. 2(c)).

The compounds eluting between 20 min and 30 min have shown the presence of molecular ion of *m/z* 469.4047, which will correspond to the [M + H]^+^ of the isomers of triterpenoid phytosterol Lupeol acetate already described in literature^23,24^.

### *Ficus carica* Latex inhibits growth of cervical cancer cells

**Figure 3:**
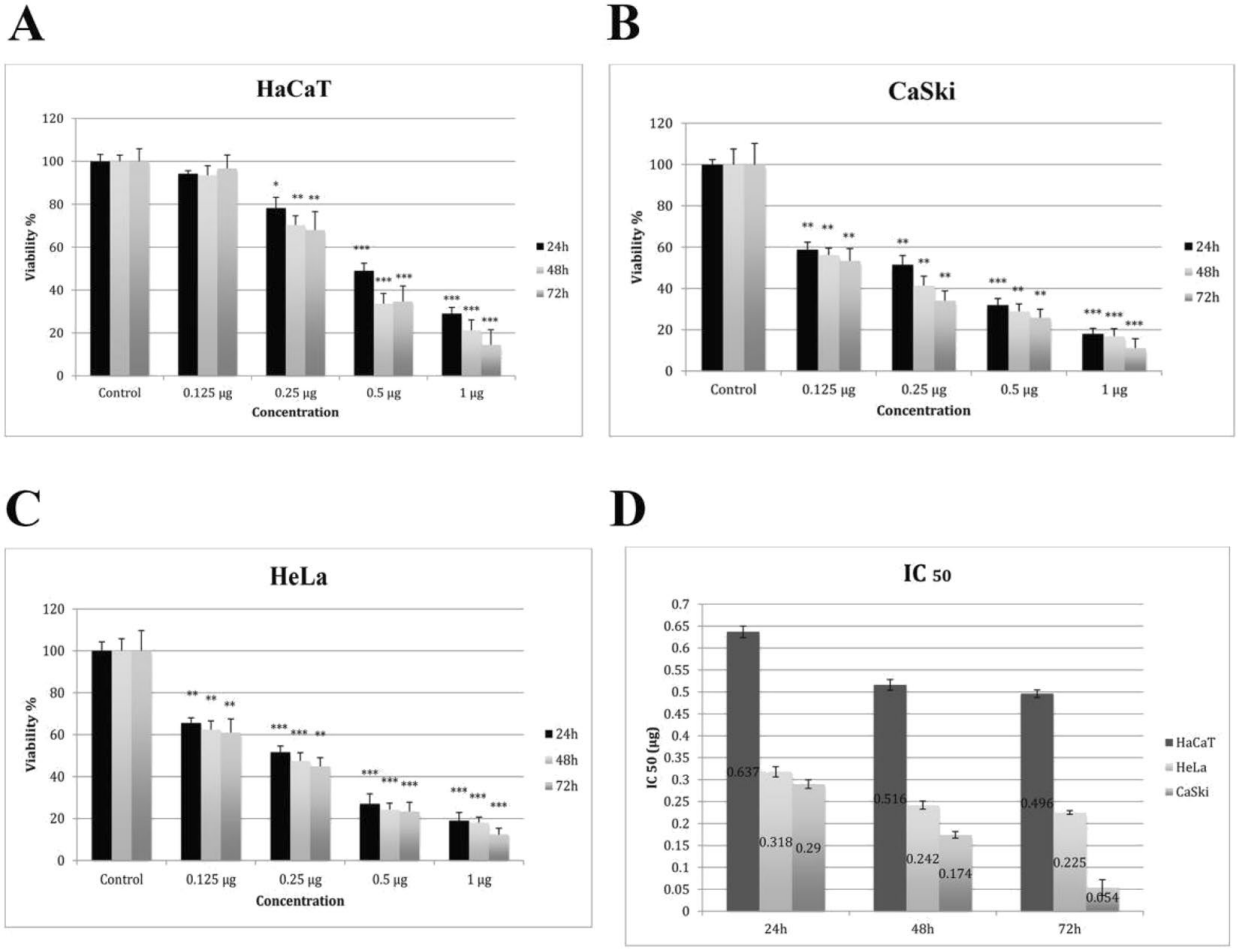
0.125 µg Fig Latex showed cytotoxicity effects on CaSki (**B**), HeLa (**C**) but not on HaCaT (**A**) cells. Cell viability was assessed using MTT assay after 24, 48 and 72 hours of treatment with fig latex. Results represent the means of at least 3 independent experiments and were normalised to control wells. Error bars indicate SEM; *p < 0.05, **p < 0.01, ***p < 0.001. (**D**) Determination of IC50 values following treatment with fig latex. Results represent the mean of at least 3.

To assess the cytotoxic activity of fig latex on cells; HeLa, CaSki and HaCaT cells were treated with different concentrations (0.125, 0.25, 0.5, and 1 $\mu$g/ml) of fig latex for 24, 48 and 72 hours. Cell viability findings suggested that, the inhibitory effect of latex on cell growth occurs in a dose-dependent but not significantly time-dependent manner (Fig. 3). As shown in Fig. 3, a certain concentration (0.125 $\mu$g/ml) of fig latex for 48 could be used to suppress cervical cancer cell proliferation “without a toxicity” effect on the normal HaCaT cells. Thus, this concentration was used to further investigate the on anti-cancer activity of fig latex against cervical cancer cells.

### *Ficus carica* latex suppresses clonogenic ability of cervical cancer cells

**Figure 4:**
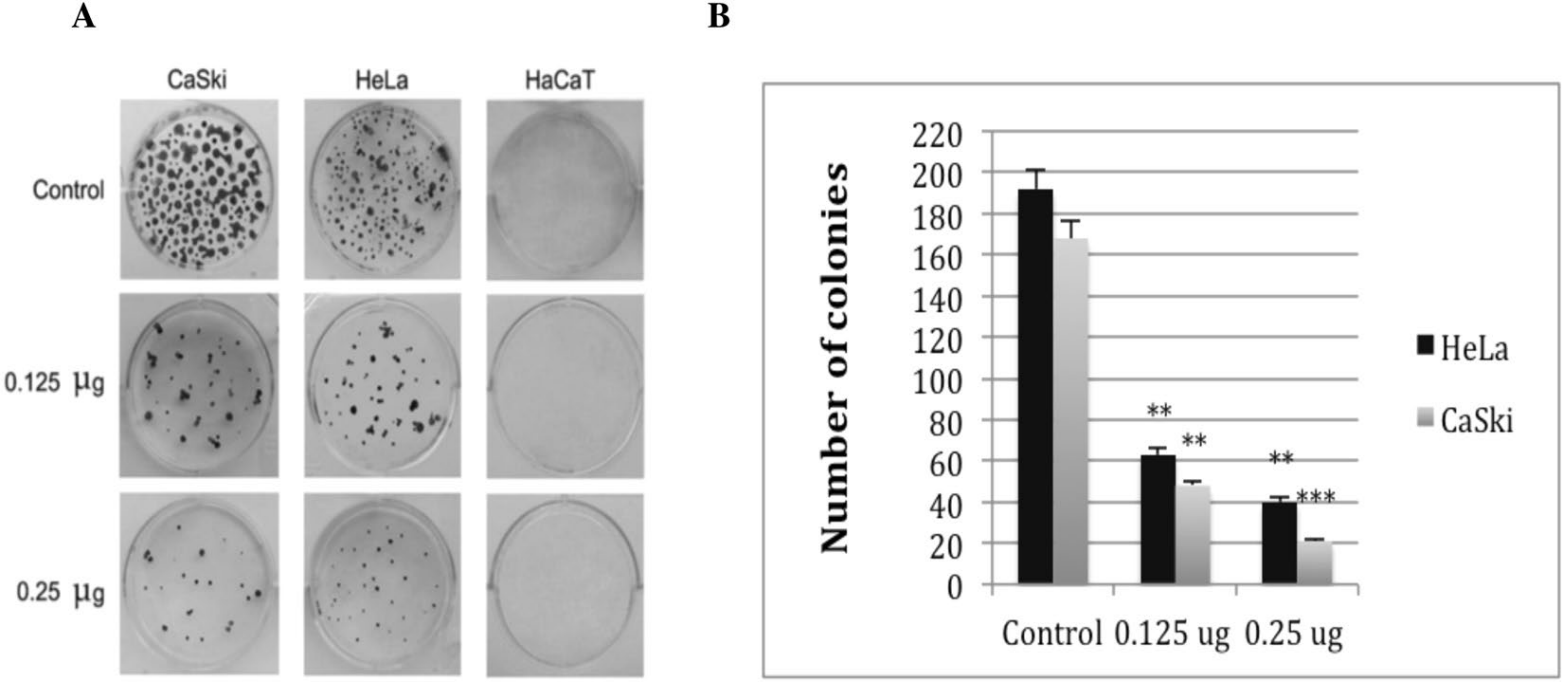
Fig latex treatment inhibits colony formation in cervical cells. (**A**) Representative colony formation images of CaSki, HeLa and HaCaT cells with and without (control) treatment. (**B**) Graphs represent the inhibitory effects of treatments measured by colony counting. Error bars show SEM; Experiments, n = 3, average of three independent experiments; *p < 0.05, **p < 0.01, ***p < 0.001.

To evaluate anti clonogenic ability of fig latex in cervical cancer, CaSki and HeLa cells were treated with non-cytotoxic concentration 0.125 $\mu$g/ml of fig latex as well as 0.25 $\mu$g/ml for 14 days. This finding suggested that fig latex is able to seize the inhibitory effect of contact inhibition driven by cervical cancer (Fig. 4).

### *Ficus carica latex* downregulates the expression of cellular proliferation marker, Ki67 protein

**Figure 5:**
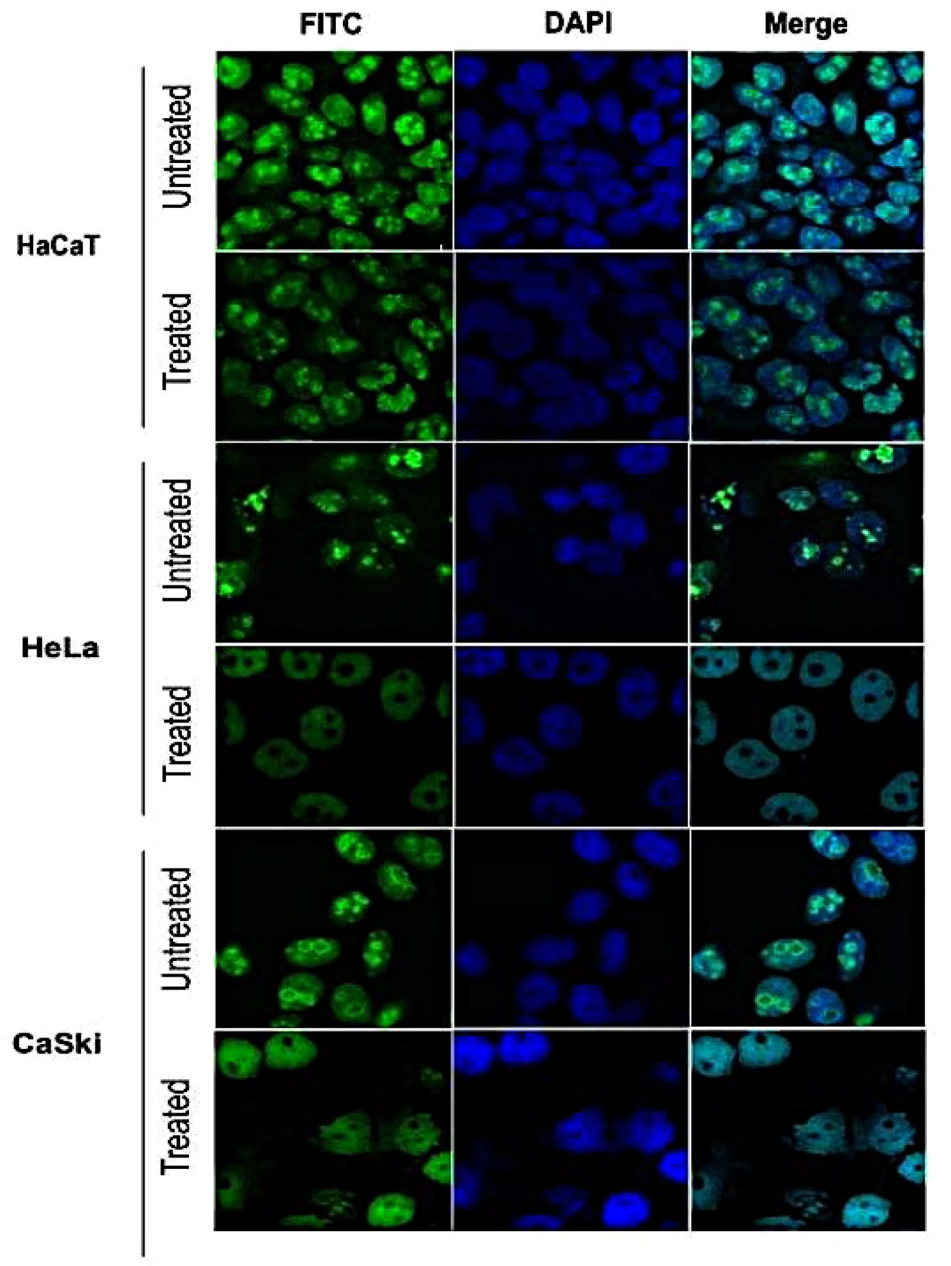
Inhibitory effects of fig latex on Ki67 proliferation protein marker on cervical cancer cells. Following treatment with 0.125 μg/ml of latex for 48 hours, the level of ki67 protein expression in HaCaT cells did not change significantly. In contrast, level of ki67 expression was downregulated significantly in treated cervical cancer cell lines. This suggests the selective inhibitory effect of fig latex on expression of Ki67 in HPV positive cells.

To investigate whether the fig latex influences cell growth in cervical cancer by targeting the Ki67 protein, control and cervical cancer cell lines were treated with a non-cytotoxic concentration (0.125 $\mu$g/ml) of fig latex for 48 hours (Fig. 5). Data from this experiment showed that ki67 protein was expressed in untreated (control) and treated HaCaT cells as well as untreated cervical cancer cell line. As shown in Fig. 5, in cervical cancer cell lines (HeLa and CaSki) treated with fig latex, Ki67 protein was depleted almost exclusively in the nucleolus. This strongly suggests that fig latex may potentially target the expression of Ki67 in cervical cancer cells to prevent cell proliferation that could ultimately play a role in the inhibition of cancer progression.

### *Ficus carica* latex suppresses cervical cancers migratory ability and alleviates invasiveness

**Figure 6:**
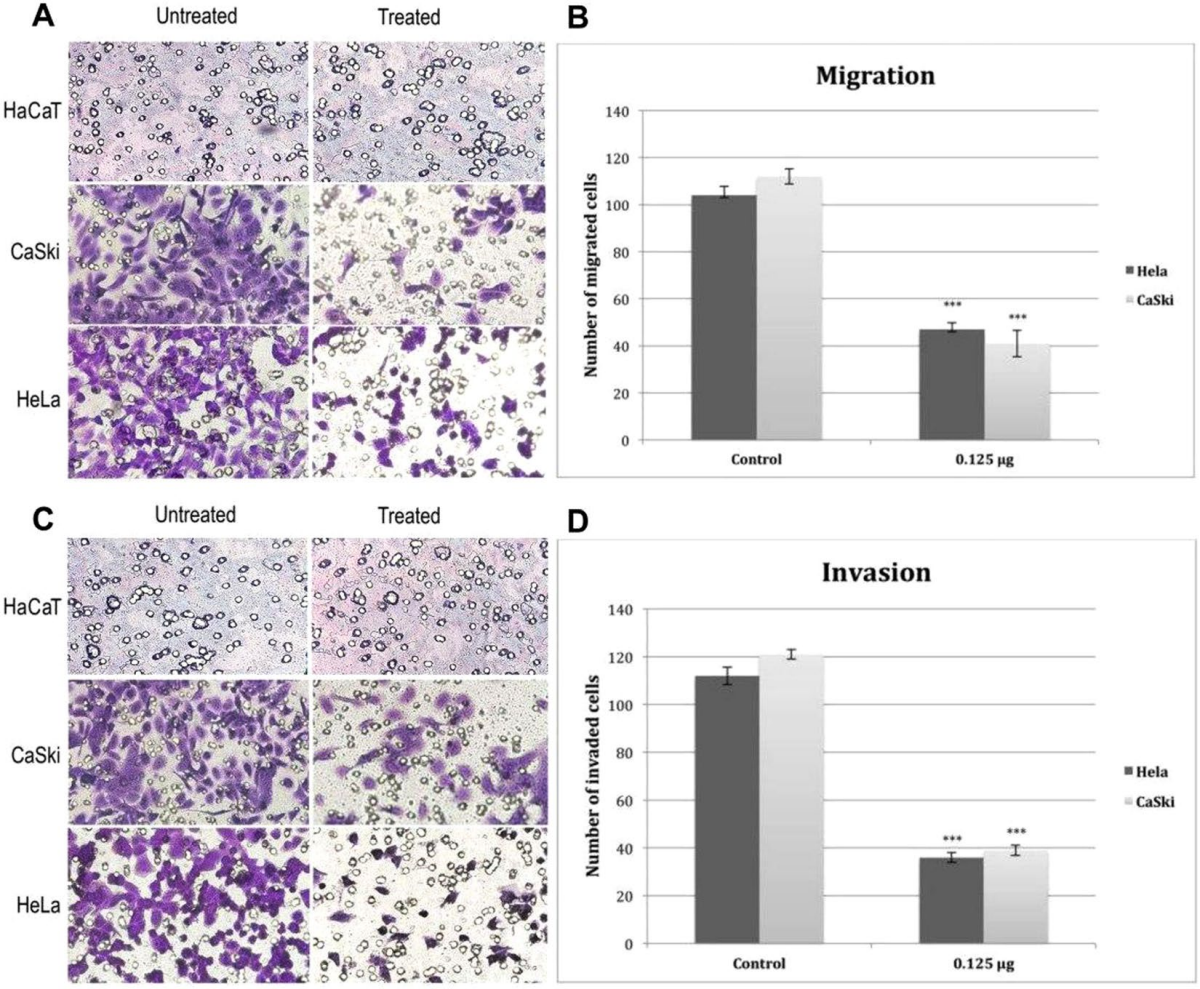
Suppression of cell migration and invasiveness of cervical cancer cells. (**A**,**C**) Representative image of Transwell migration and invasion assays respectively. (**B**,**D**) Represents the inhibitory effect of *Ficus carica* latex on the number of migrated and invaded cervical cancer cells. Error bars show SEM; Experiments, n = 6, average of six independent images; *p < 0.05, **p < 0.01, ***p < 0.001.

To investigate whether fig latex could mediate migration and invasion abilities of cervical cancer cells, CaSki and HeLa cells were treated with 0.125 $\mu$g/ml of fig latex for 48 hrs and the numbers of migrated and invaded cells towards lower chambers of transwells were counted accordingly. Findings from this experiment (Fig. 6) show that fig latex may play a significant role on the suppression of migratory capabilities of cervical cancer cells and could alleviate their invasiveness.

### *Ficus carica* latex induces cell cycle arrest in Sub G1 phase

**Figure 7:**
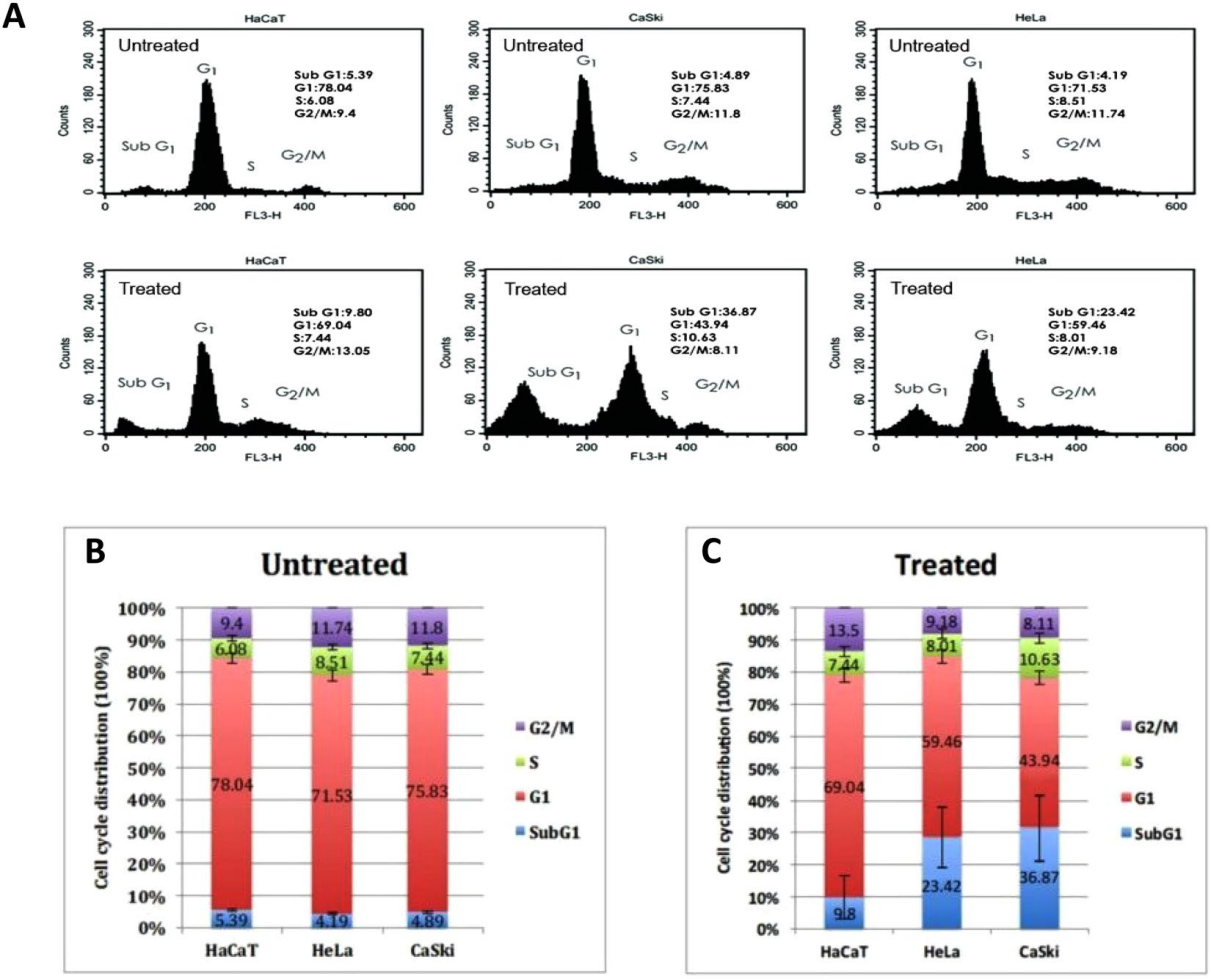
Effect of *Ficus carica* latex on cell cycle distribution of HaCaT, CaSki and HeLa cells. (**A**) Representative cell cycle analysis in treated and untreated cells. (**B**,**C**) Summarized cell cycle distribution data in untreated and treated cells respectively. Results were presented as mean (n = 3) ±SD of at least three independent experiments.

To ascertain whether the growth-inhibitory effect of fig latex on CaSki and HeLa cells is related to the cell cycle arrest, cells were treated with 0.125 $\mu$g/ml of fig latex for 48 h and the distribution of cells in Sub G1, G1, S and G2/M phases was determined and compared before and after treatment with fig latex. Data from this experiment strongly suggest that fig latex induces cell death in Sub G1 and accumulates G1 phase population in CaSki and HeLa cells. In contrast, HaCaT cells treated with fig latex showed approximately same ratio in all phases of the cell cycle (Fig. 7).

### *Ficus carica latex* downregulates the expression of HPV onco-proteins and reactivates the expression of tumour suppressor proteins

**Figure 8:**
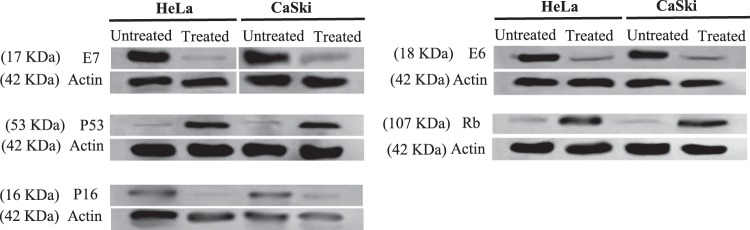
Effect of *Ficus carica* latex on the expression of HPV oncoproteins (E6 and E7), tumor suppressor proteins (P53 and Rb) and HPV diagnostic marker (P16). Equal amounts (10 µg) of protein lysate from each cell line listed were analyzed by immunoblotting with appropriate antibodies. Actin expression served as a loading control (42 kDa). *Ficus carica* latex down-regulated the expression of p16 and HPV oncproteins but upregulated the expression of tumour suppressor proteins. No significant differences were seen in the expression of actin. Un-cropped blots are available upon request.

To explore the effect of *Ficus carica* latex on HPV onco-proteins and tumor suppressor proteins, HeLa and CaSki cells were treated with 0.125 $\mu$g/ml of fig latex for 48 hrs. Expression of HPV oncoproteins (E7, E6), HPV diagnostic marker P16 and tumour suppressor proteins (P53, Rb) in treated and untreated cells were analysed using western blotting. Fig latex treatment downregulates the expression of E6, E7 and P16 and consequently upregulated the expression of tumour suppressor protein P53 and Rb in cervical cancer cells (Fig. 8). These observations suggested that fig latex possess strong anti HPV activities.

## Discussion

Although a few studies consider the distinctive medicinal features of fig latex, the mechanisms of action of the biologically active components of the *Ficus carica* species remains poorly understood. This study provides a deeper insight into the mechanisms of action and biological activities of *Ficus carica* latex on human cervical cancer cell lines expressing high risk HPVs type 16 and 18.

We report, for the first time, some of the chemical constituents of crude fig latex, aqueous supernatant and polymeric gum of fig latex using NMR/MS with the novel observation that the active anti-HPV component is likely to be lipophilic and possibly a chlorogenic/ferrulic/caffeic acid plant sterol derivative. Isolation/identification of the active from the Pet. ether fraction of the fig latex is now a priority, hampered by the limited amounts of fig latex that can be obtained from each plant. Our biological analysis confirms that the aqueous part of the latex is as effective as the crude latex and is suitable to carry out further functional studies. Using cell viability assay we have ascertained that the cytotoxic effect of latex on cell growth is dose-dependent and 0.125 μg/ml (IC50) of fig latex is the most appropriate concentration to investigate the activities of latex on cervical cancer cells. This obviates any toxic effect on the control human immortalised keratinocyte (HaCaT) cells, hence preventing any confounding data. These results corroborate the findings of previous studies on growth inhibitory effect of fig latex^22,25,26^.

We have extended our investigation on the functional activity of fig latex on cervical cancer cells to evaluate whether Ficus carica can inhibit specific properties of previously HPV related transformed cells.

Using a focus formation assay, we showed that only cervical cancer cells treated with the fig latex exit contact inhibition, a characteristic feature of cancer cells. Contact inhibition regulates cell proliferation and arrest cancer cell growth as a consequence of mechanical cell to cell interaction^27^. In addition, we have investigated the influence of fig latex treatment on the cervical cancer cell lines and our data revealed that fig latex alleviates invasiveness ability of cervical cancer cells. This finding suggests that fig latex can prevent dissemination of cancer cells into new areas and may barricade formation of metastatic tumours. Coupled with this, treatment with fig latex has allowed the cell cycle exit by inducing the cell cycle arrest in Sub G1 phase in cervical cancer cell lines. This finding demonstrates the ability of fig latex in preventing cell progression and proliferation. Cell cycle deregulation is known to be a significant element in cervical cancer carcinogenesis hence regulation of cancer cells division thorough induction of apoptosis is an efficient anti-cancer strategy^28^. Moreover, our data shows the misplacement of ki67 protein, a marker for cell proliferation^29^, in cervical cancer cells treated with the *Ficus carica* latex. This finding strongly suggests that fig latex may target the expression of Ki67 in cervical cancer cells and plays a substantial role in preventing cell proliferation that could ultimately inhibit cancer progression.

Thus, our results demonstrate a number of parameters of cell transformation (lack of contact inhibition, cell cycle arrest, invasiveness, and cell proliferation) are conferred by the Fig latex.

As mentioned before, high risk HPVs, especially type 16 and 18, can infect cervical cells and cause lesions. These lesions can persist and progress to cancer by preventing the expression of tumour suppressor proteins (P53 and Rb)^7^. Expression of E6 and E7 promotes cell invasion and metastasis, impedes apoptosis, and induces uncontrolled cellular growth and immortalization^6^. E6 interacts with P53 that is substantially contributed to the regulation of cell cycle progression via apoptosis pathways, whereas E7 stimulates hyper proliferation of the infected cells through deactivation of Rb-dependent G1/S checkpoint activation^8,30^. In addition, Rb regulates p16 via negative feedback system and therefore the Inhibition of Rb through E7 could increase the expression of p16. P16 acts as a negative regulator of proliferation in cells once the Rb has been inactivated^29^.

Nevertheless, to investigate if the effects of fig latex observed in this study were due to its impact on the expression of HPV oncoproteins, E6 and E7, we have further analysed the expression of these proteins in cervical cancer cells treated with the fig latex. Indeed, our findings from this study showed that fig latex considerably downregulates the expression level of HPV oncoproteins, E6 and E7 as well as p16 (marker for HPV infection) that was accompanied by an increase in expression level of P53 and Rb. These observations suggest that the *in vitro* effect of fig latex on the cervical cancer cell transformation might be due to remedial characteristics of the chemical constitute of fig latex on the expression of HPV oncoproteins.

In conclusion, our findings reveal that fig latex has a potential to play a role in preventing cervical cancer progression by inhibiting growth of cervical cancer cells through the upregulation of tumor suppressor proteins, p53 and pRb, which is most likely a consequence of the reduced levels of E6 and E7, respectively.

The outputs of this study provide a strong incentive to advance research in delaying HPV related cancer progression.

## Supplementary information

Supporting information
