# Supplementary material for: Biological activities of Ficus carica latex for potential therapeutics in Human Papillomavirus (HPV) related cervical cancers
Source: Sci Rep. 2019 Jan 31;9:1013. doi: 10.1038/s41598-018-37665-6 (PMC6355798; doi:10.1038/s41598-018-37665-6)
Supplement: Supplementary file 1 — Supporting information [file 41598_2018_37665_MOESM1_ESM.pdf]

Supporting information (S)

Biological activities of *Ficus carica* latex for potential therapeutics in Human Papillomavirus (HPV) related cervical cancers

Arshia Ghanbari [1], Adam Le Gresley [2], Declan Naughton [2], Nikolai Kuhnert [3], Diana Sirbu [3], G. Hossein Ashrafi [1]*

[1] Kingston University London, Cancer theme, School of Life Science, Pharmacy and Chemistry, SEC Faculty, Kingston upon Thames, KT1 2EE, London, UK

[2] Kingston University London, School of Life Science, Pharmacy and Chemistry, SEC Faculty, Kingston upon Thames, KT1 2EE, London, UK

[3] Department of Life Sciences & Chemistry Faculty of Health, Jacobs University Bremen, Campusring 8, 28759 Bremen, Germany

*** Corresponding Author: H.ashrafi@kingston.ac.uk**

Supporting information 1 (S1)

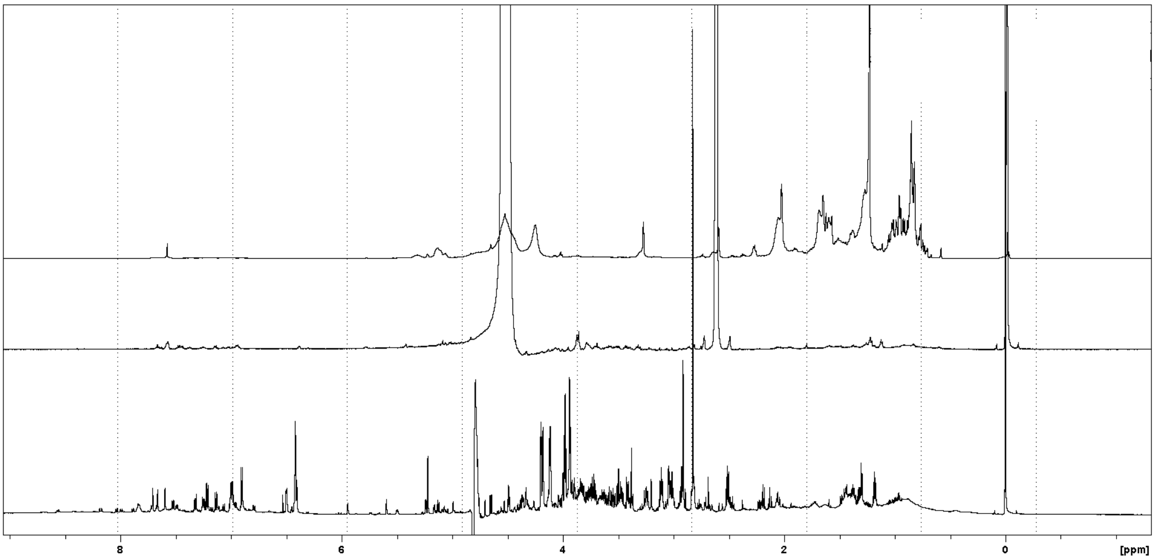

S1 ^{1}H NMR Spectra in D_2O/H_2O 90:10% of Fig latex extracts. Top – Fig Latex Gum, Middle, Fig Latex Powder, Bottom, Fig Latex Supernatant.

Supporting information 2 (S2)

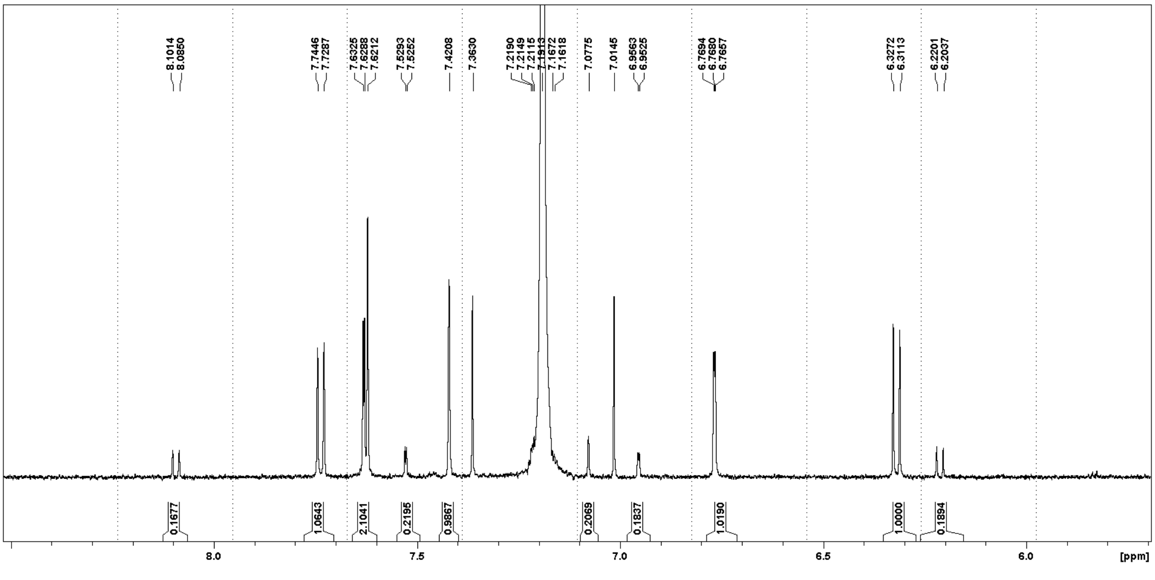

S2 ^{1}H NMR Aromatic region of petroleum ether extract of fig latex supernatant showing at least two aromatic components, suggested asferrulic, caffeic or chlorogenic acids.

Supporting information 3 (S3)

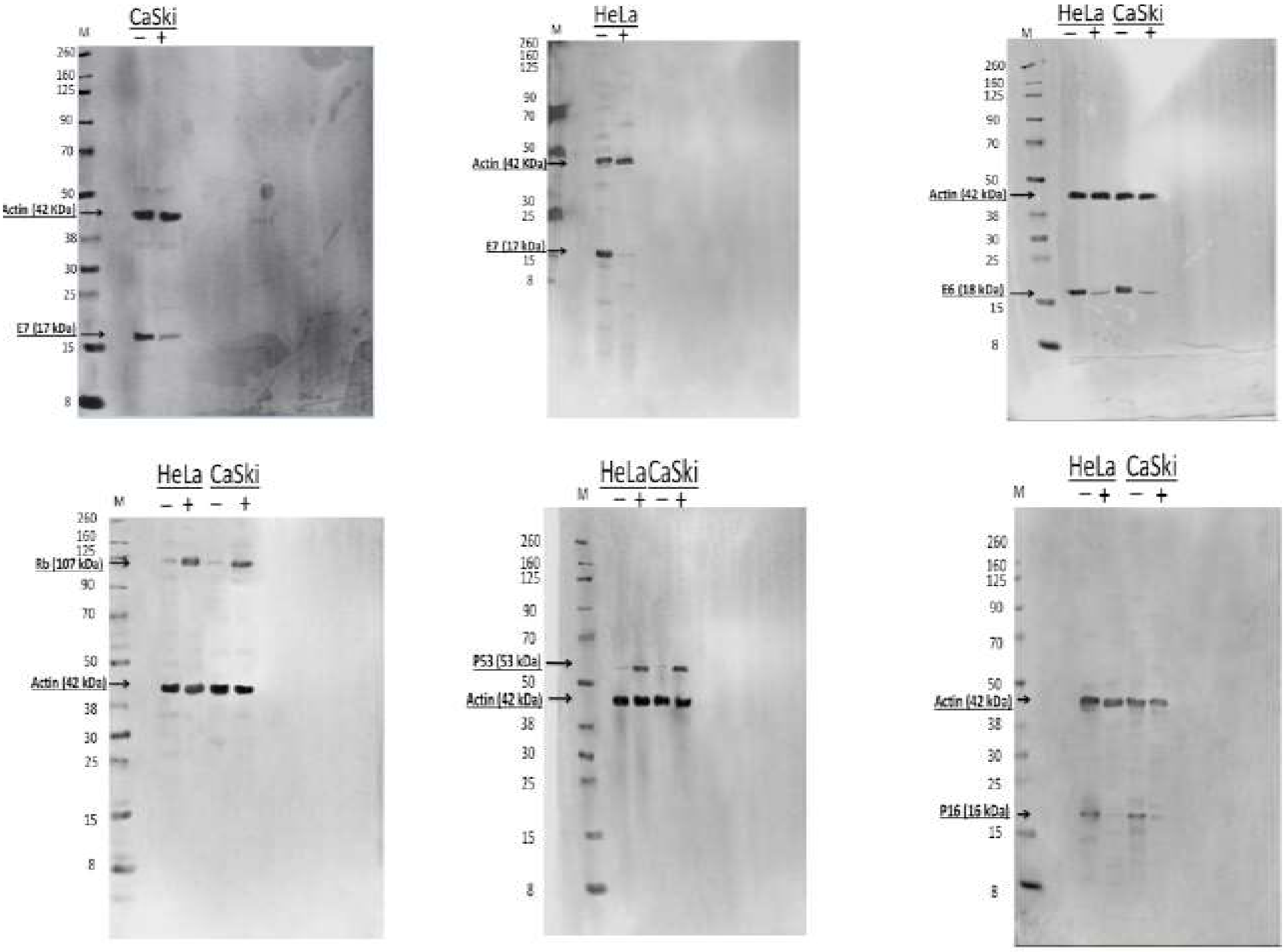

S3. Full unprocessed original scanned gels for western blots shown in figures 8.
M= Molecular weight marker
- = untreated
+ = Treated with Fig latex

Supporting information 4 (S4)

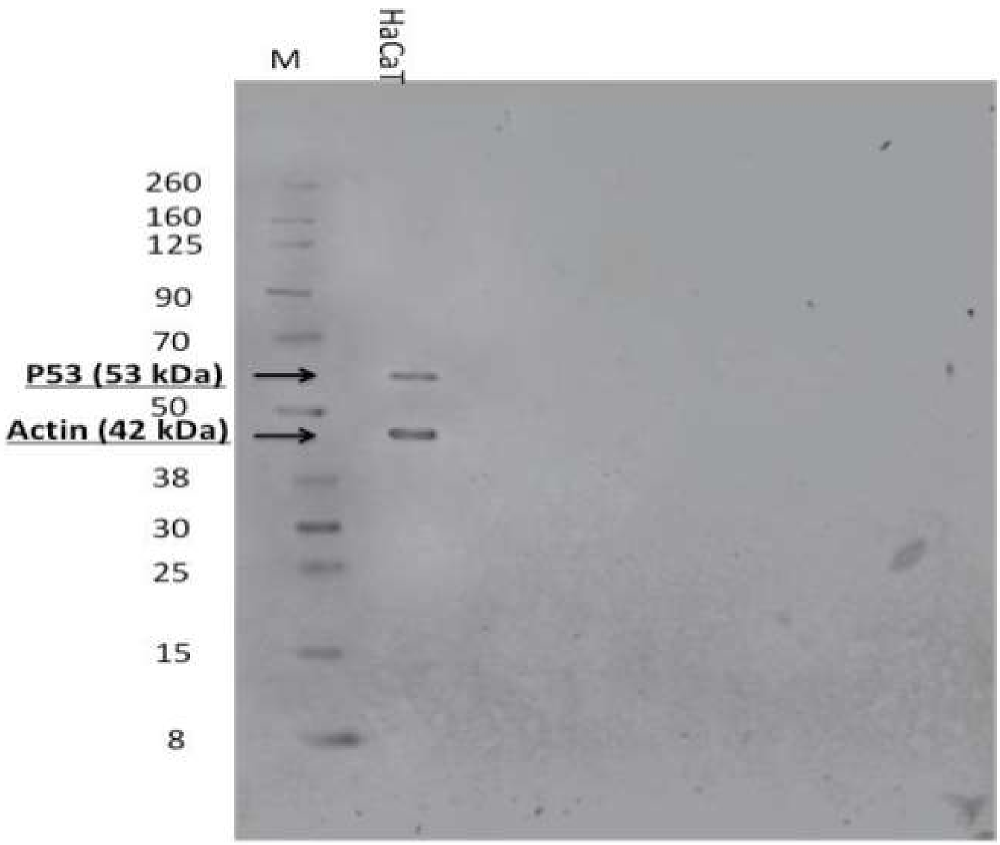

S4. Expression of p53 in HaCaT cells

Full unprocessed western blot.
M=Molecular weight marker
